# Designing Cyclic Job Rotations to Reduce the Exposure to Ergonomics Risk Factors

**DOI:** 10.3390/ijerph17031073

**Published:** 2020-02-08

**Authors:** Jose Antonio Diego-Mas

**Affiliations:** Instituto de Investigación e Innovación en Bioingeniería (I3B), Universitat Politècnica de València, Camino de Vera s/n, 46022 València, Spain

**Keywords:** job rotation, musculoskeletal disorders, evolutionary algorithms

## Abstract

Job rotation is an administrative solution to prevent work-related musculoskeletal disorders that has become widespread. However, job rotation schedules development is a complex problem. This is due to the multi-factorial character of the disorders and to the productive and organizational constraints of the real working environments. To avoid these problems, this work presents an evolutionary algorithm to generate rotation schedules in which a set of workers rotate cyclically over a small number of jobs while reducing the potential for injury. The algorithm is able to generate rotation schedules that optimize multiple ergonomics criteria by clustering the tasks into rotation groups, selecting the workers for each group, and determining the sequence of rotation of the workers to minimize the effects of fatigue. The algorithm reduces prolonged exposure to risks related to musculoskeletal injuries and simplifies the assignment of workers to different tasks in each rotation. The presented procedure can be an effective tool for the design of job-rotation schedules that prevent work-related musculoskeletal disorders while simplifying scheduled changeovers at each rotation and facilitating job monitoring.

## 1. Introduction

Work organization is defined as the way in which work is structured, managed, and processed [1]. Job rotation belongs to a group of work organizational strategies that seek to reduce workers’ exposure to ergonomic hazards by introducing variation in the tasks performed by the workers. It consists of an exchange of workers among the different workstations from time to time. A job rotation system generates benefits not only for workers but also for the company. From the workers point of view, job rotation has a positive influence as it reduces monotony [2] and boredom [3], reduces the level of stress, increases employee satisfaction [4,5] and extends the range of skills [6,7,8], retains skills for a cross-trained workforce [9], and enables workers to observe and correct deviations at an earlier production stage, which improves product quality. It also facilitates the integration of disabled workers [10], reduces fatigue and physical stress and, as a result, the incidence of work-related musculoskeletal disorders (WRMDs) [7,11,12].

The ergonomic exposure risk of a worker depends on three factors: the magnitude of the risk, exposure time, and frequency. Job rotation can act on the exposure time and frequency factors. Therefore, job rotation can be considered a preventive strategy against risk factors associated with WRMDs, such as repetitive movements, load handling, inadequate postures, or vibrations [13]. However, there is no consensus on the real effectiveness of job rotation to prevent these ergonomics risk factors [14]. While some studies reported positive effects of rotation on the prevalence of WRMDs or musculoskeletal pain [15,16], some others found undesirable effects in the long run [17,18]. The criteria used for deployment of job rotation may explain the heterogeneity of results [14]. Obtaining the expected benefits on WRMDs will only be achieved if important criteria are considered, for example, the number of workers involved, the number and duration of rotations and pauses, the use of different muscle groups in each rotation, or the physical and psychical limitations of workers [19]. On the other hand, it is important to note that rotation has no effect on the magnitude of the risk. Therefore, critical jobs that demand great effort or that expose workers to hazardous tasks must not be included in rotation programs. Reducing the risk of critical tasks before including them in a rotation program is essential to obtain efficient rotation schedules [2,20].

Job rotation reduces the incidence of musculoskeletal injuries and the level of absenteeism. Rotation fosters employee learning because it increases the possibility of assigning them to different tasks [21], and it also results in ‘firm learning’, i.e., employers learn more about their employees [22]. In general terms, companies that introduce job rotation systems are more innovative in their working methods because when the workers are rotated to other jobs, their skills and abilities can be used to improve the working tools and methods.

Developing a job rotation plan is a complex problem. The type of job rotation implemented affects the results in terms of satisfaction and productivity [23,24], and there are a lot of criteria and restrictions that must be considered simultaneously to obtain an efficient solution. For example, it is necessary to find the sequence of tasks that maximizes the variation in the muscle groups being utilized by the workers [25,26] and the optimal length of the rotation interval to reduce monotony and boredom. Simultaneously, organizational demands such as balance between job and salary must be met, and the physical and mental capacity of employees to develop the assigned tasks must be considered. Otherwise, the result may be a poorly designed job rotation plan that can adversely affect the working conditions [27].

**Figure 1:**
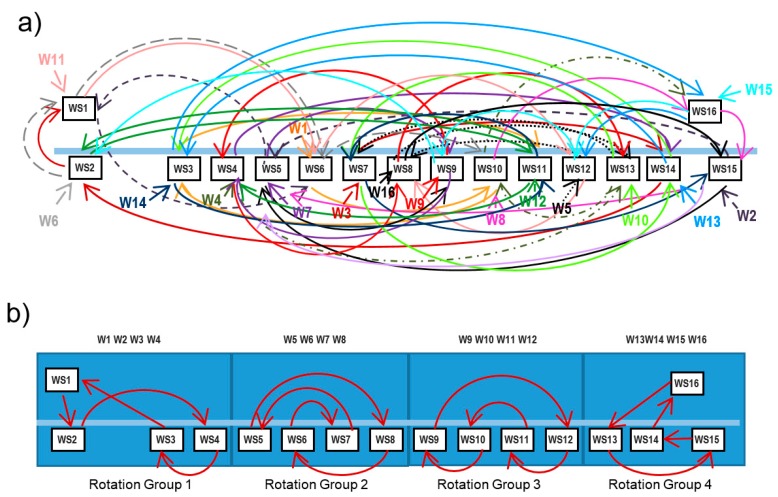
Example of a job rotation schedule with four rotations. (**a**) 16 workers rotate in 16 workstations. (**b**) Four workers rotate in four rotation groups with four workstations each one.

Several approaches to design job rotation schedules can be found in the literature [28]. Given a set of workers and workstations, obtaining an optimal rotation schedule according to some criteria is a combinatorial optimization problem. Although there are some other techniques applied to the resolution of this kind of problems, such as Integer Programming [9,10,29], one the most common ways to obtain good solutions in short times is using heuristics and metaheuristics like genetic algorithms or tabu search [25,30,31,32,33,34,35,36,37]. This paper presents RGA, an evolutionary algorithm [38] to generate job rotation schedules aimed to prevent WRMDs, that simultaneously solves important organizational problems found when rotation schedules are implemented in real environments. A rotation plan establishes the workstations that a set of workers occupy in each rotation. If the number of workstations (and workers) implied in the rotation system is large, the procedures to generate job rotation schedules generate solutions that involve complex workstations sequences that workers must follow. For example, Figure 1a shows a rotation schedule with 4 rotations in which 16 workers rotate occupying 16 workstations. Each worker is assigned to 4 different workstations and follows a different route.

Implementing a complex rotation schedule, like that shown in Figure 1a, supposes some organizational problems [39]. Each worker must follow a different and complex path, and the workers must memorize the sequence of workstations. For example, Worker 11 occupies the Workstation WS1 in the first rotation, WS6 in the second, WS12 in the third rotation, and the workstation WS8 in the last rotation. All the workers follow this kind of sequence and the movement of the workers in each rotation is a bit chaotic, leading to confusion and waste of time. On the other hand, in assembly lines it is usual that one worker (hereafter called monitor) who is responsible for a small group of workers. In some cases, the monitor may not be assigned to a particular workstation. Monitors are intended to replace workers that temporary leave his workstation, or during changeovers in job rotations in order to avoid interruptions and delays in the production process. In this work setting, implementing job rotation schedules presents difficulties for both monitors and the rest of workers. The workers assigned to one monitor follow very different sequences of workstations, making the monitoring task difficult. In each rotation, the monitor may be in different positions and workers must look for the monitor in the event of problems or substitutions. In many cases, these problems lead to abandoning rotation plans.

A commonly used strategy to implement more simple rotation schedules is grouping the workstations in several small rotation groups. For example, Figure 1b shows that the 16 workstations of the previous example have been grouped in four rotation groups. Each rotation group has four workstations and four workers assigned to them. In the example of the Figure, workstations 1 to 4 form the rotation group 1, and workers 1 to 4 are assigned to these workstations. Then, four independent rotation schedules are generated, one for each rotation group.

Although this approach avoids the organizational problems found in generating a global schedule for the complete set of workstations, it has important drawbacks. Usually, the rotations groups are created grouping workstations that are close to each other, without considering ergonomics criteria in the clustering process. In the same way, workers are assigned to rotation groups considering only organizational issues. However, to achieve the benefits expected from job rotation, it would be convenient that the movements required in the tasks developed in the workstations in the same rotation group were different. In the same way, workers should be assigned to rotation groups considering their capacities, preferences, and temporal disabilities.

To face these problems, this work proposes a different approach to create job rotation schedules. The RGA algorithm generates rotation schedules grouping the workstations to be included in the rotation plan so that a set of workers rotate cyclically over a small number of workstations. The algorithm determines how to cluster the workstations, which workers are best suited for each rotation group and the rotation sequence. This aims to: decrease the risk of musculoskeletal injuries, increase the diversification of the tasks to be performed throughout the workday, consider the possible disabilities of employees, and simplify changeovers and the monitoring task.

This paper is structured as follows: Section 2 describes the RGA algorithm, Section 3 shows a case study in an auto parts assembly line and the results are exposed in Section 4. Section 5 provides a discussion on results and, finally, Section 6 shows conclusions based on our findings.

## 2. Materials and Methods

This section describes the RGA algorithm developed to generate rotation schedules based on workstation clustering.

### 2.1. Data

To measure the quality of a rotation schedule, the suitability of the assignments of each worker in the schedule has to be measured. Therefore, it is necessary to know the requirements of each workstation and the capacities of each worker to perform them. The workstations are characterized by the body movements and the skills required to perform the tasks involved. The workers are characterized by their ability to perform the movements required by the tasks and their limitations in certain skills. In this work, the characterization of workstations and workers was based on the criteria (hereinafter called items) listed in Table 1. There are three groups of items. The Movements group contains common movements performed while developing a task [40]. The General Skills group includes general abilities commonly needed in workstations of assembly lines. Finally, the last two groups contain Communication skills and Mental abilities.

The tasks involved in the job rotation are analyzed by the personal of the Occupational Health and Safety Department of the company to determine which body movements must be performed by the worker and how often the worker performs them. This analysis provides a numerical value for the movement items required to perform the tasks at each workstation according to the scoring system shown in Table 2.

Additionally, the workers’ capacity to perform the movements listed in the Movement items is scored. The score is 0 if the worker has no limitations to perform the movement, 1 if the worker has a low limitation in the movement, 2 if the limitation is high, and 3 if the worker is unable to perform the movement. Medical advice is needed to assign scores to the items of the workers depending on their capacity to perform each movement and to decide if some worker has some limitation to perform some activity.

The score of the items of the General and Mental and Communication skills for jobs and workers is qualitative. If a skill is required to perform a particular task, the corresponding item is scored as “Necessary”, otherwise it is scored as “Not Necessary”. The workers´ skills items take the values “With Limitation” or “Without limitation”, depending on whether or not the worker has limited capacity to perform the skill specified by the item.

### 2.2. The RGA

The algorithm proposed in this paper follows the general principles of evolutionary algorithms [41]. A set of structures representing solutions to the problem are evolved performing a stochastic guided search for the best solution. Initially, a set of solutions is created (usually in a random way) coding each solution by a vector or a matrix (chromosome or individual). The set of solutions is called population. In the RGA algorithm, each chromosome represents a complete rotation schedule establishing the workstation that each worker should occupy in each rotation. The population is then transformed to obtain a new set of solutions. The new solutions are obtained applying operators such as “selection”, “crossover”, or “mutation” which transform the solutions combining or modifying the chromosomes of the current population. These operators act according to the fitness of each solution. The fitness of a solution is obtained by means of an objective function previously defined. The new population undergoes the same process, and this procedure is repeated until a stop condition is reached.

This section describes the procedures specifically developed for the RGA, i.e., the solution encoding, how the initial population of chromosomes is generated, the fitness function used to measure the quality of a rotation schedule, the penalties for unwanted solutions, the way to select the individuals to form the next generation, and the individuals’ crossover and mutation processes.

#### 2.2.1. Solution Encoding and Initial Population

**Figure 2:**
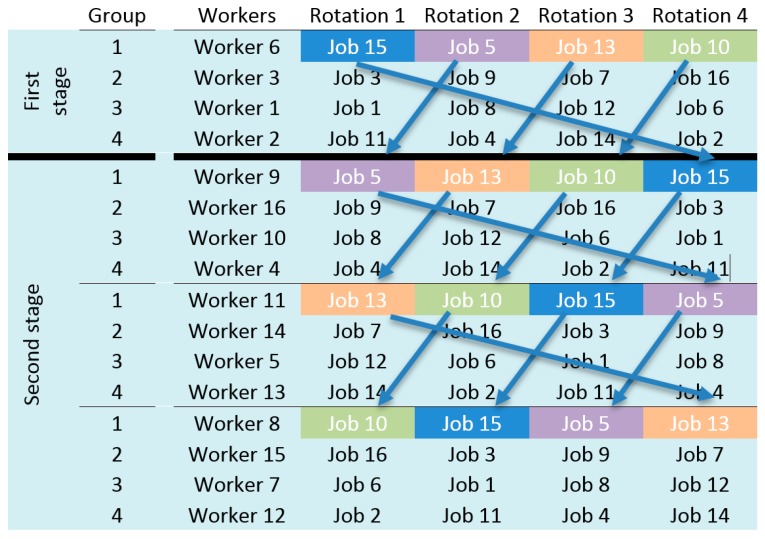
Solution encoding and procedure for the generation of individuals.

RGA generates an initial population of solutions of size n. Each solution is a job rotation schedule that is encoded using a matrix of size n_w_ × 1 + n_r_, where n_w_ is the number of workers involved in the schedule and n_r_ is the number of rotations during a working day (Figure 2). The number of workers must coincide with the number of workstations to be occupied and must be divisible by n_r_.

The objective of RGA is that each group of workers (rotation group) rotates cyclically over a small number of workstations. The number of rotation groups (n_g_) is obtained by dividing the number of workers (n_w_) by the number of rotations (n_r_). That is, n_g_ is the number of rotation groups in each schedule, each group consisting of n_w_/n_g_ workers and workstations.

In the matrix used to encode the solutions (Figure 2), the column Workers indicates a worker, and the remaining columns of the row indicate the workstation that this worker will occupy in each rotation. The first row of the matrix contains a worker of the first rotation group, the second row a worker of the second rotation group, and so on until row n_g_. Row n_g_ + 1 contains a worker of the first rotation group, row n_g_ + 2 a worker of the second group, and so on, i.e., row i indicates the jobs belonging to rotation group i − {n_g_ × Integer_part [(i−1)/n_g_]} assigned to a worker in each rotation. For example, in the case of a job schedule with 4 rotations, 16 workers and 16 workstations (Figure 2), rows 1, 5, 9, and 13 contain the workers and workstations of rotation group 1; rows 2, 6, 10, and 14 those of group 2; rows 3, 7, 11, and 15 those of group 3; and rows 4, 8, 12, and 16 those of group 4.

The creation of one solution is performed in two stages. In the first stage, the rotations of the first n_g_ rows are generated. These rows contain a worker from each rotation group. n_g_ workers are randomly assigned to the cells in the column Workers. A workstation is also randomly assigned to the remaining cells of the rows. Since the number of cells is n_g_ × n_r_, the workstations will not be repeated within the same row. In the second stage, the rest of the workers are randomly assigned to the first column of rows n_g_ + 1 to n_w_. The workers in the same rotation group should occupy the same set of workstations in different rotations.

**Figure 3:**
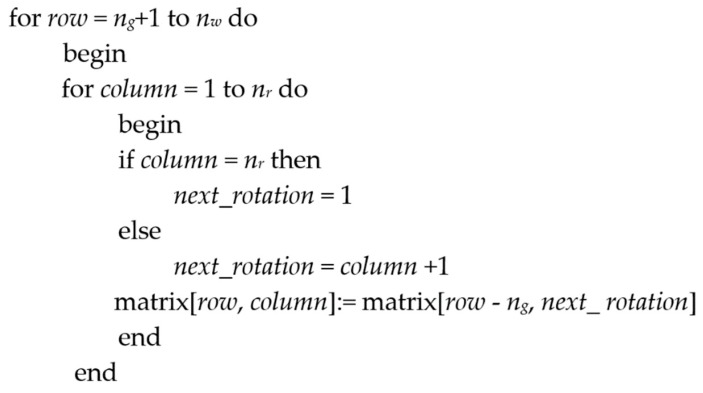
Pseudo-code of the procedure for the assignment extension.

In the first stage, the set of workstations assigned to each group is defined, and one worker is assigned to each rotation group. Each new worker in a rotation group will be assigned the tasks that the previous worker performed in the previous rotations to complete the matrix (see Figure 1). This procedure to complete a solution is called “Assignment Extension”. Figure 3 shows the pseudo-code of the procedure for the assignment extension.

In the example of Figure 2, the first row, generated in the first stage, indicates that worker 6 is assigned successively in each rotation the jobs 15, 5, 13, and 10. All these jobs belong to group 1. To complete the jobs in the fourth row, also corresponding to rotation group 1, the same sequence of jobs as in the first row are assigned, but now displaced 1 element. Therefore, worker 9 will be assigned job 5 in the first rotation, then job 13, job 10, and finally, job 15 in the fourth rotation. In this way, workers in each rotation group will rotate cyclically over the same set of jobs.

This procedure is repeated until the n individuals that conform the initial population are generated. The n parameter depends greatly on characteristics of the problem to be solved. In our tests, good results have been obtained using a population size of 50 to obtain rotation schedules for 16 workstations and 4 rotations.

#### 2.2.2. Measuring the Fitness of the Rotation Schedules

Each solution in the population represents a rotation schedule. To obtain the fitness of one solution, the suitability of the schedule that it represents must be measured. The suitability of the schedule can be obtained measuring the suitability of the assignments of the workers in each rotation. If the worker i has been assigned to the workstation y in the rotation z, the suitability of this assignment can be measured multiplying the scores of the items of the job y by the scores of the items of the worker i. Extending this procedure to all the rotations and workers, we can measure the fitness (E) of a rotation schedule (Equation (1)). The scores of a workstation are proportional to the need of performing some movements or activities in the task performed in this workstation, while the scores of a worker are inversely proportional to the capacity of the worker to perform these movements or activities. Therefore, the value of E, calculated by means of Equation (1), will be lower for the most suitable schedules. The lower the E, the better the schedule:

(1) $$E=\overset{n_{w}}{\underset{i=1}{\sum }}\overset{n_{r}}{\underset{r=1}{\sum }}\overset{n_{i}}{\underset{j=1}{\sum }}I_{j}*w_{j}^{i}\left(r\right)*t_{j}\left(m^{i}\left(r\right)\right)*d_{r}.$$

In Equation (1), n_w_, n_r_, and n_i_ are the number of workers, rotations, and items considered. The coefficient I_j_ represents the relative importance of the item j with respect to the rest of the items, tj(mi(r)) is the score of the item j of the job allocated to the worker i in the rotation r and d_r_ is the duration of the rotation r.

w^i^_j_(r) is the score of the item j of worker i in rotation r. The capacity of a worker to perform a particular movement must be recalculated after each rotation, considering the effects of the jobs performed in earlier rotations, i.e., cumulative effect of fatigue on the muscle groups involved. To do this, the values of the workers’ movement items w^i^_j_(r) are recalculated for each rotation using the Equation (2) that considers the movements already performed by the worker in preceding rotations. If a worker had been assigned to a workstation requiring, for example, elbow flexion, in a rotation before the current rotation, the score of the worker’s item for this movement would increase according to the physical effort and duration of the task. This will reduce the probability of assigning this worker to a job that requires elbow flexion in the next rotations:

(2) $$w_{j}^{i}\left(r\right)=w_{j}^{i}+\frac{1}{r_{d}}*\overset{r-1}{\underset{\begin{array}{c} h=1\\ t_{j}\left(m^{i}\left(h\right)\right)>\mathrm{th} \end{array}}{\sum }}\frac{t_{j}\left(m^{i}\left(h\right)\right)*l_{h}}{e_{h}}.$$

Equation (2) calculates the values of the movement items of the workers in each rotation (w^i^_j_(r) is the value of the item j of the worker i in the rotation r). This equation considers that the effort required at each workstation is given by the values of the workstation’s movement items. How much the movement items of the workers will change in the rotation r depends on the duration of the tasks developed in previous rotations (l_h_ is the duration of the rotation h performed previously to rotation r) and on the time elapsed since these tasks were performed (e_h_ is the time elapsed between the end of the rotation h and the beginning of the rotation r). For example, the longer a task that requires elbow flexion has been performed in previous rotations, the more the elbow flexion item will increase in the current rotation. In the same way, the longer the time elapsed since the end of the tasks requiring elbow flexion, the lower the increment of the elbow flexion item. In this way, the chance of being assigned to a workstation that requires elbow flexion will gradually increase as time passes. To calculate the time elapsed between rotations (Equation (3)), the existence of breaks or pauses is considered. In Equation (3), t_h_,_r_ is the break time between the current rotation r and a previous rotation h, and l_g_ is the duration of the rotation g performed between h and r:

(3) $$e_{h}=\left\{\begin{array}{c} t_{h,r}+\overset{r-1}{\underset{g=h+1}{\sum }}l_{g};h<r-1\\ 1\;;h=r-1 \end{array}\right\}.$$

Equation (2) increases the scores of the items of a worker only if the items of the workstations of previous rotations are bigger than a threshold value (th). Items with scores below this threshold value do not cause any fatigue effects on the worker and, therefore, the scores of the worker items are not modified. The parameter r_d_ regulates the size of the effect of tasks performed in preceding rotations on the items of the worker in later rotations. The bigger the value of r_d_, the lower the effect.

#### 2.2.3. Penalties

The fitness function shown in Equation (1) does not penalize the assignment of a worker to the same workplace in successive rotations. However, to achieve the benefits expected from job rotation, the worker should be assigned to different workstations. In this way, different muscle groups are used in each rotation and the monotony is reduced. To avoid assigning a worker to the same workstation in consecutive rotations, the parameter t_max_ is defined as the maximum consecutive time that a worker can be assigned to a workstation. The solutions in the population are revised to check if some worker has been assigned to the same workstation for a time longer than t_max_. In this case, the solution is penalized by increasing its fitness value, hence reducing its chances to survive or reproduce.

On the other hand, the unwanted worker-job assignments are identified in this stage of the RGA. This will prevent certain workers with limited capacities from being assigned to workstations that require these skills. The values of the General, Mental and Communication skills items for the workstations and for the workers assigned to them are compared. To prevent solutions with unwanted assignments from passing to the next generation, their fitness values (E) are increased (penalized), thus reducing their probability of being selected as survivors. Finally, the solutions encoding unwanted assignments due to organizational or medical reasons are also penalized.

#### 2.2.4. Selection

At this stage, all the solutions in the population have been assigned a fitness score and a roulette wheel selection operator [42] is used to select survival solutions (solutions that pass to the next population). In this selection process, the probability of a solution of being selected to survive is proportional to the suitability of the job rotation schedule that the solution represents. In RGA, the fitness of a solution, calculated by means of Equation (1), will be lower for the most suitable schedules. Therefore, the lower the E, the greater the probability of the solution of being selected.

Roulette wheel selection operator does not guarantee that the best solutions of the population are always selected as survivor. This can lead to loosing good solutions. To avoid this, the best solutions of the population are always selected as survivors (elitism). E_r_ is the number of the best solutions retained for the next generation (elitism rate).

The new generation of solutions is compounded of survivors, elite individuals, and new individuals obtained by crossover of solutions of the previous generation. The number of new individuals obtained by crossover is n·p_c_, being n the population size and p_c_ a parameter named crossover probability. The number of survivors selected by the roulette wheel selection method from the previous generation is n·(1 − p_c_) − E_r_.

#### 2.2.5. Crossover Operator

Solutions involved in the crossover procedure are also selected by roulette wheel selection. n·p_c_ pairs of individuals (parents) are selected from the population. Each pair is crossed over and generates a new individual (offspring). The offspring passes directly to the new generation. The crossover procedure is performed as follows:

1. Two ‘parent’ solutions are randomly selected from the population and called Parent A and Parent B.
2. One of the first n_g_ rows of the offspring that have not yet been selected (hereafter named row i) is randomly selected.
3. Each cell in the row i of the offspring is assigned the same value as the equivalent cell in the row i of parent A. If the value corresponding to a cell has already been assigned to any other cell of the offspring, then the cell will remain empty and will be marked as ‘to be completed later’.
4. Parent A will be now Parent B, and Parent B will be now Parent A. Steps 2, 3, and 4 are repeated until all the n_g_ rows of the offspring have been selected.
5. The cells of the offspring marked as ‘to be completed later’ are randomly assigned the jobs that have not yet been assigned to any other cell.
6. After obtaining the first n_g_ rows of the offspring, the remaining rows are generated by assignment extension (Section 2.2.1).
7. One of the parents is randomly selected and the values of the column 1 (workers) are copied in the column 1 of the offspring.

Crossover operator considers only the first n_g_ rows of the ‘parent’ individuals and creates only the first n_g_ rows of the offspring. Then, the complete offspring is obtained using the assignment extension procedure (Section 2.2.1).

**Figure 4:**
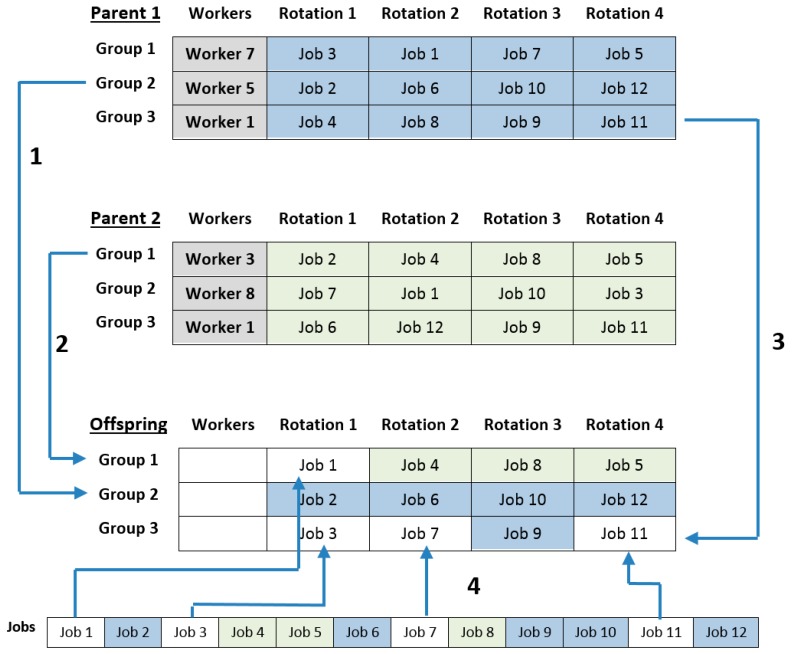
Example of generation of offspring through crossover of two parents. The numbers 1 to 4 represent the sequence followed to generate the offspring.

Figure 4 shows an example of a crossover operation between two solutions with three rotation groups. In the first step, row 2 of the Parent 1 is copied into the row 2 of the offspring. Then, in the second step, the row 1 of the Parent 2 is copied into the row 1 of the offspring, except the job of the first rotation (Job 2), which had been previously assigned in step 1. This cell is marked as ‘to be completed later’. The procedure is repeated with the row 3 of the Parent 1 in the third step. Finally, the marked cells are randomly completed with jobs not assigned yet in the last step.

#### 2.2.6. Mutation Operator

Mutation acts over randomly selected solutions. The number of individuals that will mutate is n·p_m_, where n is the population size and p_m_ is a parameter (mutation probability). The mutation procedure is divided in two phases. The first phase deals with the assignment of workstations to each rotation, and the second deals with the assignment of workers to each group. The procedure is repeated n·p_m_ times:

1. One individual is randomly selected from the population.
2. A rotation group j is randomly selected.
3. Two rows, between 1 and ng, are randomly selected (i1, i2).
4. The jobs assigned to cells (i1, j) and (i2, j) are swapped.
5. The assignment is extended (Section 2.2.1).
6. Two workers of column 1 are randomly swapped.

## 3. Case Study

This section describes the procedure used to verify the ability of the RGA algorithm to generate cyclic job rotation schedules that minimize the effects of repetitive movements while simplifying scheduled changeovers at each rotation. To do this, RGA was used to design a cyclic rotation schedule for a set of 16 workplaces and workers in a spare parts assembly line.

Cyclic rotation schedules are preferable from an organizational point of view. However, it is not the same from an ergonomics point of view. All the solutions in the RGA’s initial population are cyclic rotation schedules. On the other hand, crossover and mutation in RGA are closed operators (they always produce valid cyclic schedules) and then, all the solutions in the population of RGA are always cyclic rotation schedules. Therefore, the solution space in which RGA searches for a solution to the problem is formed by all the possible cyclic rotation schedules. This solution space is a subset of all the possible schedules (including all the no-cyclic schedules). As a result, from an ergonomics point of view, the quality of the best schedule in the RGA´s solution space will be equal or worse than that of the best schedule in the global solution space including no-cyclic schedules.

RGA’s approach looks for a compromise between ergonomics and organizational criteria to generate rotation schedules. To check this compromise, the problem under consideration was solved using RGA to obtain the best cyclic rotation schedule. Then, a modified version of the algorithm (RGAm) was used to obtain the best no-cyclic job rotation schedule. Finally, the scores of the fitness function of both solutions were compared. To create a schedule in RGAm, the workers are randomly assigned to the workstations without creating rotation groups. In the same way, the crossover operator does not consider rotation groups and creates offspring from parents in an unrestricted way.

In the analyzed spare parts assembly line, there was an eight-hour workday. RGA was used to design a cyclic rotation schedule with four rotations; the first three rotations with a duration of 2 h, and the last one of 1 h. There was one-hour break for lunch between the second and the third rotations. All the 16 workstations belonged to same line and the distances between them allow for quick changeovers. The 16 workers had skills to perform the task required at any of the workstations.

### 3.1. Scores of the Workers and the Workstations

The movements and the skills needed to perform the task in each workstation of the assembly line were analyzed. Scores between 0 and 3 were assigned to each item of the movements group (Table 1) depending on the frequency of each movement (Table 2). In the same way, the medical staff of the manufacturing plant collaborated to assign scores to the items of the workers depending on their capacity to perform each movement and to decide if some worker had some limitation to perform some activity. Table 3 shows the scores of the items of the movements group for each workstation and for each worker. The skills and capacities needed in each workstation and the skills and limitations of the workers were matched to determine which workers should not be assigned to some jobs (penalized assignments). Table 4 shows the penalized assignments.

### 3.2. Parameters Used in the RGA

The duration of the rotations was set to two hours for the first, second, and third rotation and one hour for the fourth. t_max_ (the maximum consecutive time that a worker can be assigned to a workstation) was set to 2 h. In this way, the solutions that assigned a worker to the same workstation in two consecutive rotations were penalized. The coefficients I_1_ to I_18_ were set to 1 (see Equation (1)). These coefficients represent the relative importance of each item with respect to the rest of the items. In this case, all of them were considered to have the same level of importance.

Equation (2) increases the scores of the items of a worker after each rotation if the items of the workstations of previous rotations are bigger than a threshold value (th). In the same Equation, the parameter r_d_ regulates the size of the effect of tasks performed in preceding rotations on the items of the worker in later rotations. Considering the advice of the medical staff, the value of the parameter th was set to 1.5 and r_d_ was set to 3.

Some tests were performed to establish the best combination of the rest of the parameters of RGA. In general, RGA is a robust algorithm and slight changes in the parameters do not affect the results. After running the algorithm several times, the population size (n) was set to 50, the crossover probability (p_c_) was set to 0.6, and the mutation probability (p_m_) to 0.3. The elitism rate (E_r_) was set to 1.

## 4. Results

RGA was run 10 times using the scores shown in Table 3 and the penalizations shown in Table 4. Each run stopped after 10,000 iterations. Then, the modified version of the algorithm (RGAm) was run 10 times using the same scores and parameters to obtain the best no-cyclic job rotation schedule. The algorithms were executed in a personal computer with a 2.7 GHz CPU of 4 cores and 32 GB RAM. The mean execution time for the 10 runs was 8.36 min for the RGA and 6.24 min for the RGAm algorithm.

The results of each execution of RGA and RGAm are shown in Table 5; Table 6, respectively. The first column of the tables shows the run number, the second (Iteration) shows the generation in which the best schedule was found, and the third (Best Fitness), the fitness value of the best schedule. The fourth column (Workers mean value) indicates the mean contribution of the assignments of each worker to the total fitness of the best solution. Finally, the last column shows the standard deviation of the contribution of the assignments of each worker to the fitness of the best solution. The values of the last column indicate if the tasks have been evenly assigned to the workers. A high value in this column means that some workers have been assigned to tougher workstations (with high scores in the movements items) while others performs lighter ones. Therefore, lower values are preferable in this column.

The algorithms were able to obtain feasible solutions in all the runs and the restrictions of the problem were satisfied. The fitness value of the best schedule provided by the RGA 492.80, while the value of best solution obtained by RGAm was 477.33. The workers mean value was 31.84 for RGA and 29.96 for RGAm. On average, RGA obtained the best fitness in iteration 3643 and RGAm in iteration 4861.90.

RGA found the best schedule in Run 3 (Table 7). The first rotation group was formed by the workstations 15, 5, 13, and 10 and the workers 6, 9, 11, and 8. The group 2 consisted of workstations 3, 9, 7, 16 and workers 3, 16, 14, and 15. Group 3 included workstations 1, 8, 12, and 6 and workers 1, 10, 5, and 7. Finally, group 4 consisted of workstations 2, 4, 13, and 12 and workers 2, 4, 13, and 12. This solution met the constraints imposed on the problem and the workers were allocated in different workstations in each rotation. This was true even for disabled workers whose limitations prevented them from performing several movements or activities. On the contrary, in the best solution generated by RGAm (Table 8), the worker 12 was assigned to the same workstation in two non-consecutive rotations (rotation 1 and 3).

The last column in the Table 7; Table 8 indicates the contribution of the assignments of each worker to the total fitness of the solution. The balance in the assignments of the workers was very similar in the best solution found by both RGA and RGAm. The best RGA solution was slightly more balanced than the best RGAm solution. In the best RGA solution, the largest imbalance was between worker 9, with an average cost of 24.23, and worker 14 with an average cost of 47.49. In the best RGAm solution, the largest difference was found between worker 7, with an average cost of 20.04, and worker 14, with an average cost of 45.55. Worker 14 was the worker with more cost in both solutions. Worker 14 had limitations in some movements and skills and could not occupy several workstations, which meant less possibilities for both algorithms when trying to achieve more favorable and less imbalanced assignments compared to those of other workers. The same happened with worker 12, whose cost was 46 in the best RGA solution and 42.04 in the best RGAm solution.

## 5. Discussion

The RGA algorithm generates rotation schedules grouping the workstations to be included in the rotation plan in such a way that a set of workers rotate cyclically over a small number of workstations. The algorithm determines how to cluster the workstations, which workers are best suited for each rotation group, and the rotation sequence. Solutions obtained using RGA may decrease the risk of musculoskeletal injuries, increase the diversification of the tasks to be performed throughout the workday, and consider the possible disabilities of employees. In addition, RGA simplifies changeovers between rotations, avoiding delays in the production process, while facilitating the monitoring tasks. The computational experiences show the ability of the RGA algorithm for solving the problem of the generation of rotation schedules considering simultaneously multiple criteria and constraints creating rotation groups. Under these conditions, the algorithm provides good results with small computation time. In the case under study, the model was able to obtain solutions in which workers rotate cyclically over four workstations while minimizing movement repetitiveness and may balance the cumulative effect of fatigue and stress.

The space of solutions of the current problem, in which only cyclic schedules are considered valid solutions, is a subgroup of the space of solutions to the problem without workstations clustering. This suggests that, necessarily, the value of the fitness function of the best solution in the RGA solution space will be greater than or equal to the value of the best solution without job clustering. In fact, the RGAm version of the algorithm, was able to generate a solution to our test problem with a slightly lower value of the objective function than RGA. To check if the best solution obtained using RGA is significantly worse from an ergonomic point of view than that obtained using RGAm, it would be convenient to know the optimum solution to the problem, but it is not possible to obtain that value in an analytical way. Therefore, we used another reference to compare both solutions. One thousand random rotations schedules were generated as solutions to our test problem and the values of the fitness function were calculated. The mean value of the fitness function was 649.22 (SD 16.06) and the mean cost by the worker was 40.58 (SD 15.92).

The fitness value of the best rotation schedule obtained using RGA was 492.80 and using RGAm was 477.33. Therefore, the fitness of the best cyclic schedule was 24.09% better than the mean fitness of random schedules, and the fitness of the best no-cyclic schedule was 26.47% better. The difference between both algorithms compared to the random allocation is 2.38%. Therefore, although the best cyclic rotation schedule is slightly worse than the best no-cyclic schedule from the ergonomics point of view, the difference is small, and it is compensated by the benefits of a better work organization.

**Figure 5:**
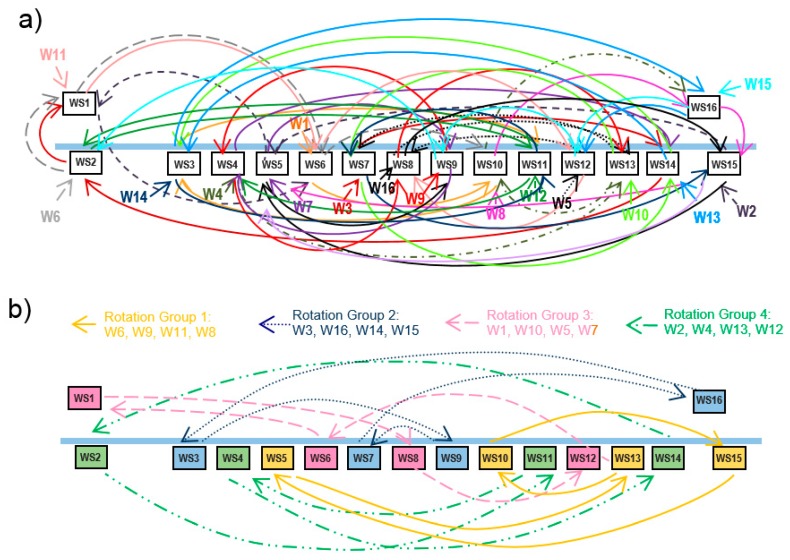
Paths of the workers in the solutions provided by the algorithms (**a**) RGAm and (**b**) RGA.

The Figure 5 shows the workers’ paths during a working day for the best cyclic solution (Figure 5a) and the best no-cyclic solution (Figure 5b). The no-cyclic solution shows some organizational problems such as the diversity and complexity of the routes followed by the workers or the chaotic movement of the workers during changeovers. On the other hand, the best cyclic solution simplifies the workers’ paths during the working day. The rotation groups allow knowing the workstations around which the workers would rotate, enabling a better organization and monitorization of the work. The problems derived from the implementation of a solution like that shown in Figure 5a, entail, in many occasions, an abandonment of the rotation plan. Hence, the organizational benefits obtained with the use of the RGA seem to make up the increase of the value of the fitness function.

The ergonomics criteria considered in this work are mainly intended to reduce the exposure of workers to repetitive movements. However, other criteria can be included in the process to reduce the exposure of the workers to other musculoskeletal disorders risk factors or to prevent musculoskeletal pain or disability. One of the advantages of our proposal is the additive model used in the fitness function of RGA. This additive model gives the possibility of including new factors and criteria to be considered to generate the job rotation schedules. Each new criterion can be included, adding a new term to the fitness function with a weighting coefficient to consider its relative importance with respect to other criteria. In this way, for example, the Nordic Musculoskeletal Questionnaire [43] could be used to consider the workers musculoskeletal pain, the NIOSH lifting equation [44] to take into account the effects of handling loads or the Rapid Entire Body Assessment (REBA) [45] for awkward postures. The results of the application of each of these evaluating tools could be included as items to be considered and added to the evaluating function with their corresponding weighting coefficients.

## 6. Conclusions

In this study, we have presented a new procedure to generate cyclic job rotations schedules. RGA generates rotation schedules that group workstations in rotation groups so that a set of workers rotate cyclically in a small number of jobs. The algorithm determines how to cluster the jobs, which workers are best suited for each group, and the sequence of rotation. The solutions obtained by this procedure minimize movement repetitiveness, diversify the content of the tasks performed by the workers during the workday, take the limitations of workers into consideration, and balance the cumulative effect of fatigue and stress. In addition, job clustering simplifies changeovers between rotations, avoiding delays in the production process, while facilitating the monitoring of the workers.

Although, from an ergonomic point of view, no-cyclic schedules performs better, our results shows that cyclic schedules are only slightly worse than t no-cyclic schedules and that this difference is compensated by the benefits of a better work organization.

## Figures and Tables

**Table 1:** Criteria employed to characterize workers and workstations. The skills and capacity to perform the movements of the workers are matched with those required by the jobs.

Movements	General Skills	Mental and Communication Skills
Arm abduction	Standing	Reasoning
Arm extension	Sitting	Taking complex decision
Arm flexion	Walking	Responsibility
Elbow flexion	Climbing	Cooperation
Neck extension	Coordinating movements	Attention
Neck flexion	Applying force standing	Initiative
Neck turning	Applying force in movement	Autonomy
Neck lateralization	Driving vehicles	Long distance vision
Shoulder raising	Working at height	Color vision
Trunk flexion	Using personal protection equipment	Hearing
Trunk rotation	Staying in confined/restricted spaces	Locating direction of sound
Trunk extension		Tactile sensitivity
Trunk lateralization		Smelling/tasting
Pinching with fingers		Writing
Hand flexion		Speaking
Hand extension		Using a keyboard
Pronation/Supination of hands		Using a mouse
Radial/Cubital deviation of hands		

**Table 2:** Score assigned to the workstations depending on the frequency of movements that must be performed.

Frequency (Movements/Minute)	Score
0	0
1–2	1
3–7	2
> 7	3

**Table 3:** Scores of the items of the “Movements” group for the 16 workstation and workers. The first number in each cell is the workstation score, the second one is the worker score.

	1	2	3	4	5	6	7	8	9	10	11	12	13	14	15	16
Arm-abduction	1|0	2|0	2|0	2|0	1|0	0|0	1|0	1|0	1|0	1|0	1|0	2|0	1|0	1|0	2|0	1|0
Arm-extension	0|0	1|0	1|0	1|0	1|0	1|0	1|0	0|0	0|0	1|0	1|0	2|0	1|0	1|0	1|0	1|0
Arm-flexion	3|0	2|0	2|0	2|0	1|0	2|0	2|0	2|0	3|0	2|0	2|0	2|0	3|0	2|0	2|0	2|0
Elbow-flexion	3|0	1|0	1|0	1|0	1|0	2|0	2|0	2|0	3|0	2|0	2|0	1|0	2|0	2|0	2|0	2|0
Neck-extension	0|0	0|0	0|0	0|0	1|0	0|0	0|0	0|0	0|0	0|0	0|0	0|0	0|0	0|2	0|0	0|0
Neck-flexion	3|0	3|0	3|0	3|0	1|0	2|0	1|0	2|0	3|0	3|0	3|0	2|0	3|0	3|1	3|0	3|0
Neck-turning	2|0	1|0	1|0	1|0	1|0	1|0	1|0	2|0	3|0	3|0	2|0	2|0	2|0	3|1	2|0	2|0
Neck-lat.	0|0	0|0	0|0	0|0	1|0	0|0	0|0	0|0	0|0	0|0	0|0	0|0	0|0	0|2	0|0	0|0
Shoulder raising	0|0	1|0	1|0	1|0	1|0	0|0	0|0	0|0	0|0	0|0	0|0	0|2	0|0	0|1	0|0	0|0
Pinching	2|0	2|0	2|0	2|0	1|0	1|0	1|0	2|0	2|0	2|0	2|0	2|0	2|0	2|0	2|0	2|0
Hand-flexion	3|0	1|0	1|0	1|0	2|0	1|0	2|0	2|0	2|0	2|0	3|0	2|0	2|0	2|0	1|0	2|0
Hand-extension	1|0	0|0	0|0	0|0	1|0	0|0	1|0	1|0	1|0	0|0	1|0	0|0	1|0	1|0	1|0	1|0
Hand-turning	1|0	1|0	1|0	1|0	1|0	2|0	1|0	2|0	2|0	1|0	2|0	1|0	2|0	2|0	2|0	2|0
Hand-lat.	1|0	1|0	1|0	1|0	1|0	0|0	1|0	1|0	1|0	1|0	0|0	0|0	1|0	1|0	1|0	1|0
Trunk-flexion	2|0	1|0	1|0	1|0	2|0	1|0	1|0	1|0	2|0	2|0	1|0	1|2	2|0	2|1	1|0	1|0
Trunk-extension	0|0	0|0	0|0	0|0	0|0	0|0	0|0	0|0	0|0	0|0	0|0	0|0	0|0	0|0	0|0	0|0
Trunk-rotation	1|0	0|0	0|0	0|0	1|0	1|0	1|0	1|0	1|0	2|0	0|0	1|3	1|0	2|2	0|0	0|0
Trunk-lat.	0|0	0|0	0|0	0|0	1|0	0|0	1|0	0|0	0|0	1|0	0|0	0|3	0|0	1|1	0|0	0|0
Legs-flexion	2|0	0|0	0|0	0|0	1|0	0|0	1|0	0|0	0|0	1|0	0|0	0|2	1|0	1|0	1|2	0|0

**Table 4:** Penalized assignments as a result of limitations of the workers’ capacities and job requirements.

Workstation	1	2	3	4	5	6	7	8	9	10	11	12	13	14	15	16
Worker 12	●				●	●	●	●	●	●		●	●		●	●
Worker 13	●				●	●	●		●	●						
Worker 14	●				●	●										
Worker 15	●					●										

**Table 5:** Results of the RGA algorithm.

Run	Iteration	Best Fitness	Workers Mean Value	Standard Deviation of Workers Mean Value
1	2969	492.83	31.62	9.61
2	2857	492.83	32.05	11.14
3	2353	492.80	32.30	14.26
4	3071	496.40	32.17	9.87
5	8276	492.83	32.09	12.32
6	5363	492.83	31.30	11.19
7	1397	494.37	31.40	10.41
8	1888	492.83	31.69	11.86
9	6777	494.37	31.43	9.97
10	1479	493.65	32.35	11.82
**Mean values**	**3643**	**493.57**	**31.84**	**11.24**

**Table 6:** Result of the RGAm algorithm.

Runs	Iteration	Best Fitness	Workers Mean Value	Standard Deviation of Workers Mean Value
1	3141	477.77	29.86	8.03
2	6153	479.32	29.96	7.87
3	3230	480.71	30.05	7.92
4	7071	479.54	29.97	7.49
5	2545	479.93	30.00	7.86
6	6345	477.33	29.83	7.15
7	8697	478.52	29.91	6.72
8	4479	479.73	29.98	7.56
9	5421	480.07	30.00	6.35
10	1537	480.92	30.06	6.41
**Mean values**	**4861.90**	**479.38**	**29.96**	**7.33**

**Table 7:** Best solution generated by RGA.

Cycle	Workers	Rotation 1	Rotation 2	Rotation 3	Rotation 4	Cost
1	Worker 6	Workstation 15	Workstation 5	Workstation 13	Workstation 10	24.37
2	Worker 3	Workstation 3	Workstation 9	Workstation 7	Workstation 16	24.70
3	Worker 1	Workstation 1	Workstation 8	Workstation 12	Workstation 6	32.25
4	Worker 2	Workstation 11	Workstation 4	Workstation 14	Workstation 2	30.89
1	Worker 9	Workstation 5	Workstation 13	Workstation 10	Workstation 15	24.23
2	Worker 16	Workstation 9	Workstation 7	Workstation 16	Workstation 3	29.22
3	Worker 10	Workstation 8	Workstation 12	Workstation 6	Workstation 1	26.20
4	Worker 4	Workstation 4	Workstation 14	Workstation 2	Workstation 11	26.72
1	Worker 11	Workstation 13	Workstation 10	Workstation 15	Workstation 5	36.32
2	Worker 14	Workstation 7	Workstation 16	Workstation 3	Workstation 9	47.49
3	Worker 5	Workstation 12	Workstation 6	Workstation 1	Workstation 8	26.12
4	Worker 13	Workstation 14	Workstation 2	Workstation 11	Workstation 4	31.74
1	Worker 8	Workstation 10	Workstation 15	Workstation 5	Workstation 13	27.03
2	Worker 15	Workstation 16	Workstation 3	Workstation 9	Workstation 7	31.37
3	Worker 7	Workstation 6	Workstation 1	Workstation 8	Workstation 12	28.01
4	Worker 12	Workstation 2	Workstation 11	Workstation 4	Workstation 14	46.06
				Average cost		30.80
				Standard deviation		7.07
				Total fitness		492.80

**Table 8:** Best solution generated by RGAm.

Workers	Rotation 1	Rotation 2	Rotation 3	Rotation 4	Cost
Worker 1	Workstation 6	Workstation 10	Workstation 3	Workstation 11	23.48
Worker 2	Workstation 15	Workstation 5	Workstation 1	Workstation 6	21.48
Worker 3	Workstation 7	Workstation 14	Workstation 2	Workstation 1	22.53
Worker 4	Workstation 4	Workstation 13	Workstation 10	Workstation 16	32.98
Worker 5	Workstation 12	Workstation 8	Workstation 13	Workstation 7	29.92
Worker 6	Workstation 2	Workstation 1	Workstation 6	Workstation 10	25.62
Worker 7	Workstation 5	Workstation 9	Workstation 4	Workstation 14	20.04
Worker 8	Workstation 10	Workstation 16	Workstation 15	Workstation 5	32.72
Worker 9	Workstation 9	Workstation 4	Workstation 8	Workstation 13	34.42
Worker 10	Workstation 13	Workstation 7	Workstation 14	Workstation 3	28.46
Worker 11	Workstation 1	Workstation 6	Workstation 12	Workstation 8	27.39
Worker 12	Workstation 11	Workstation 2	Workstation 11	Workstation 4	42.04
Worker 13	Workstation 14	Workstation 3	Workstation 16	Workstation 12	31.43
Worker 14	Workstation 3	Workstation 11	Workstation 7	Workstation 15	45.55
Worker 15	Workstation 16	Workstation 12	Workstation 9	Workstation 2	34.31
Worker 16	Workstation 8	Workstation 15	Workstation 5	Workstation 9	24.96
			Average cost		29.83
		Standard deviation			7.14
			Total fitness		477.33
